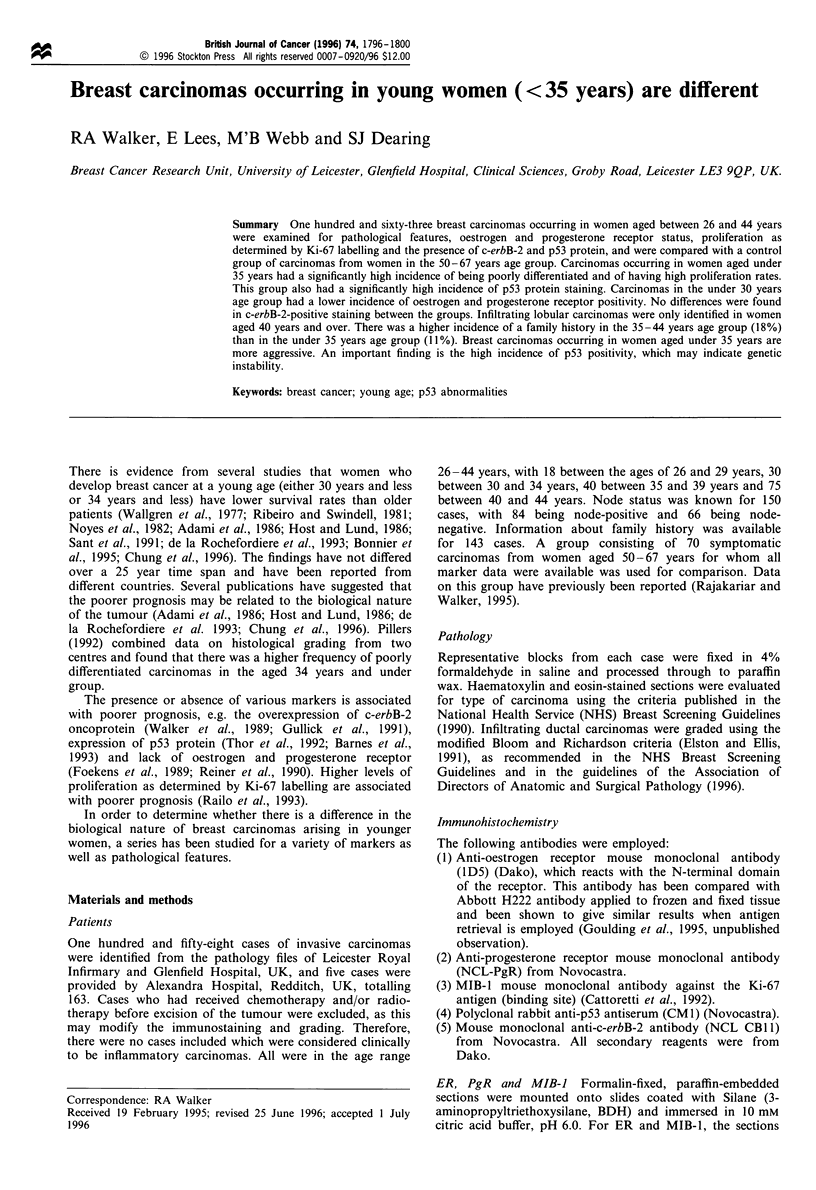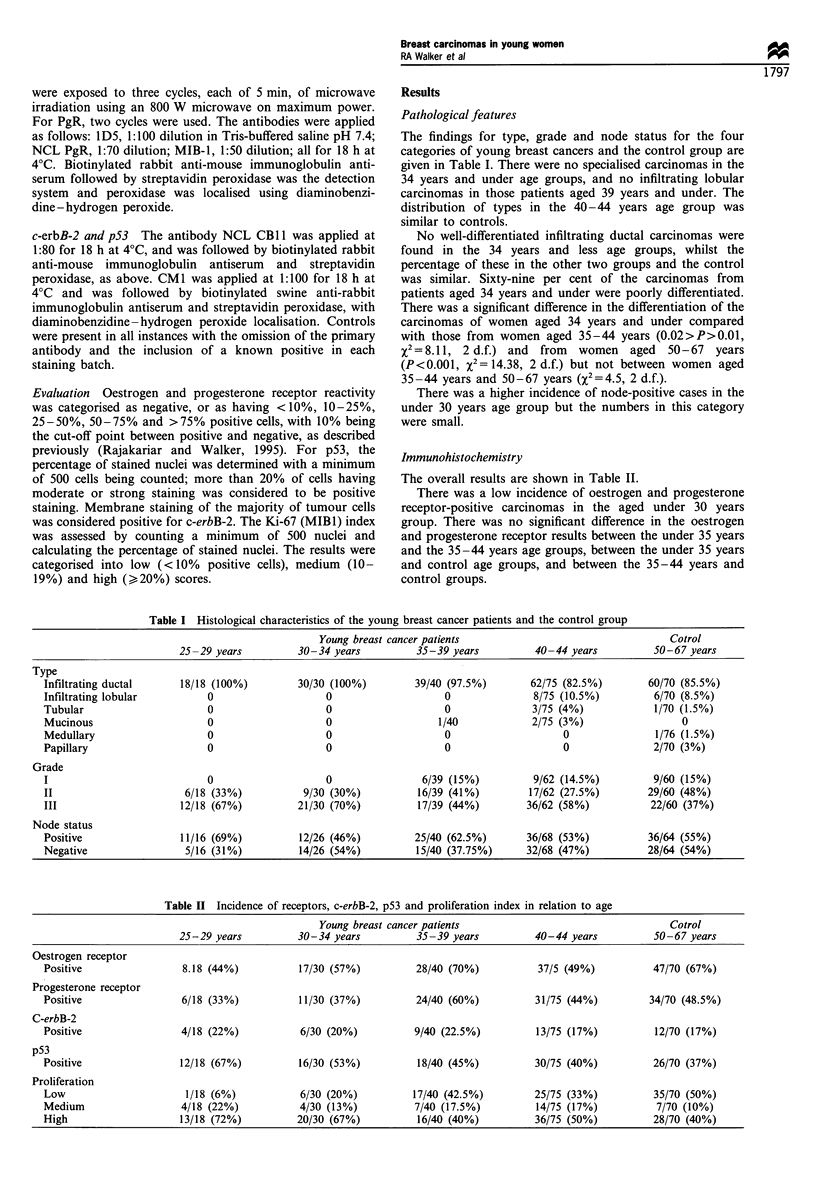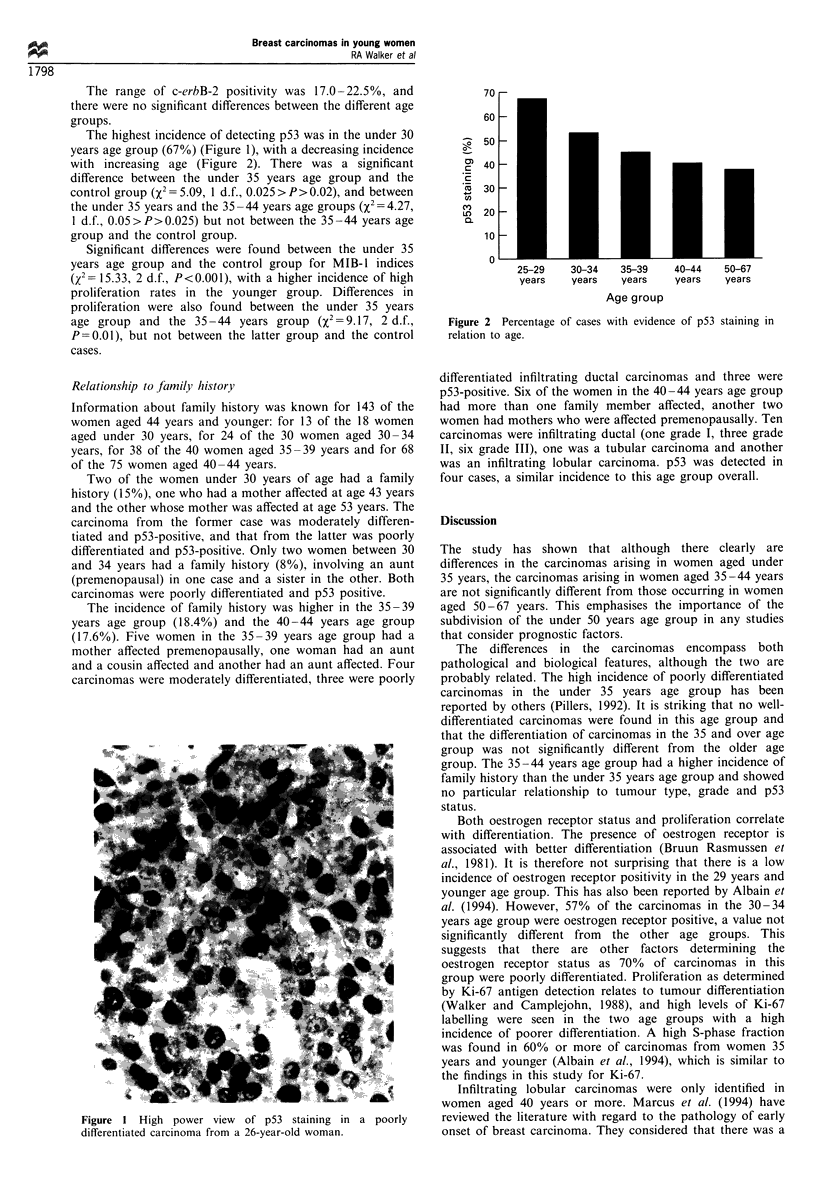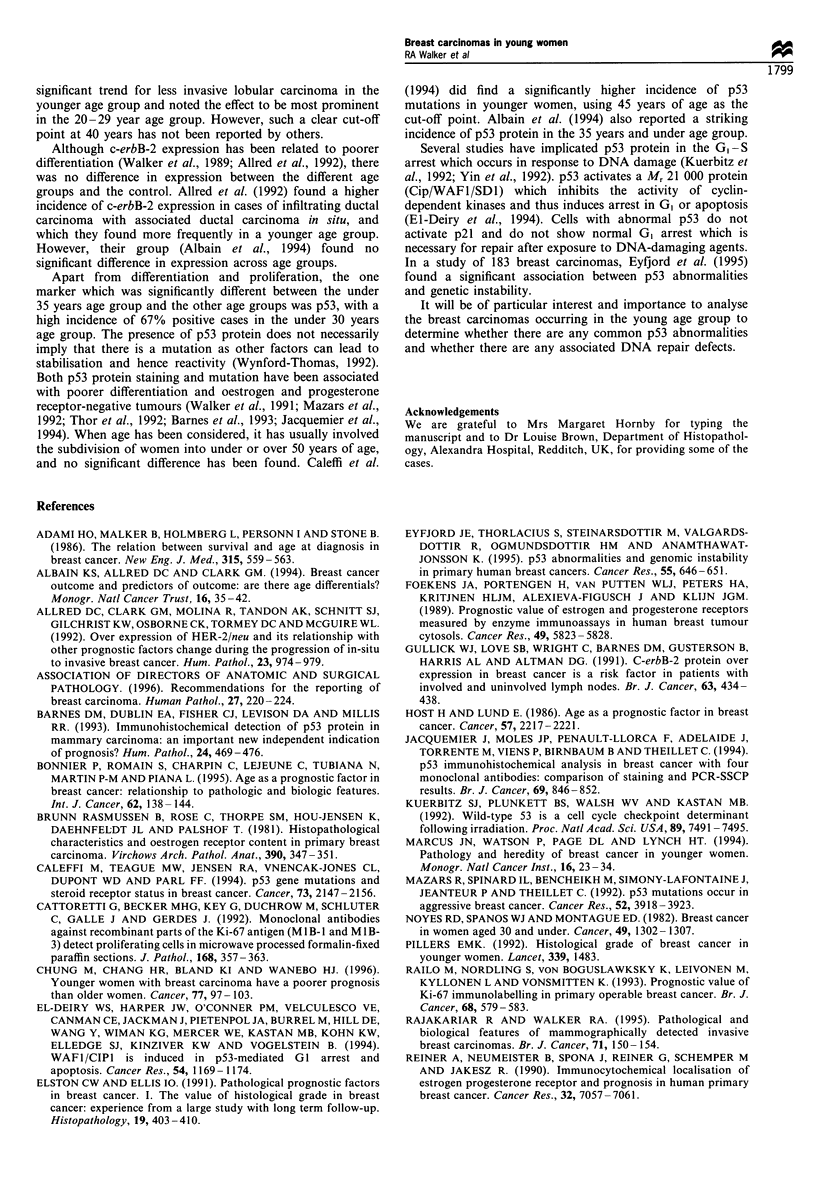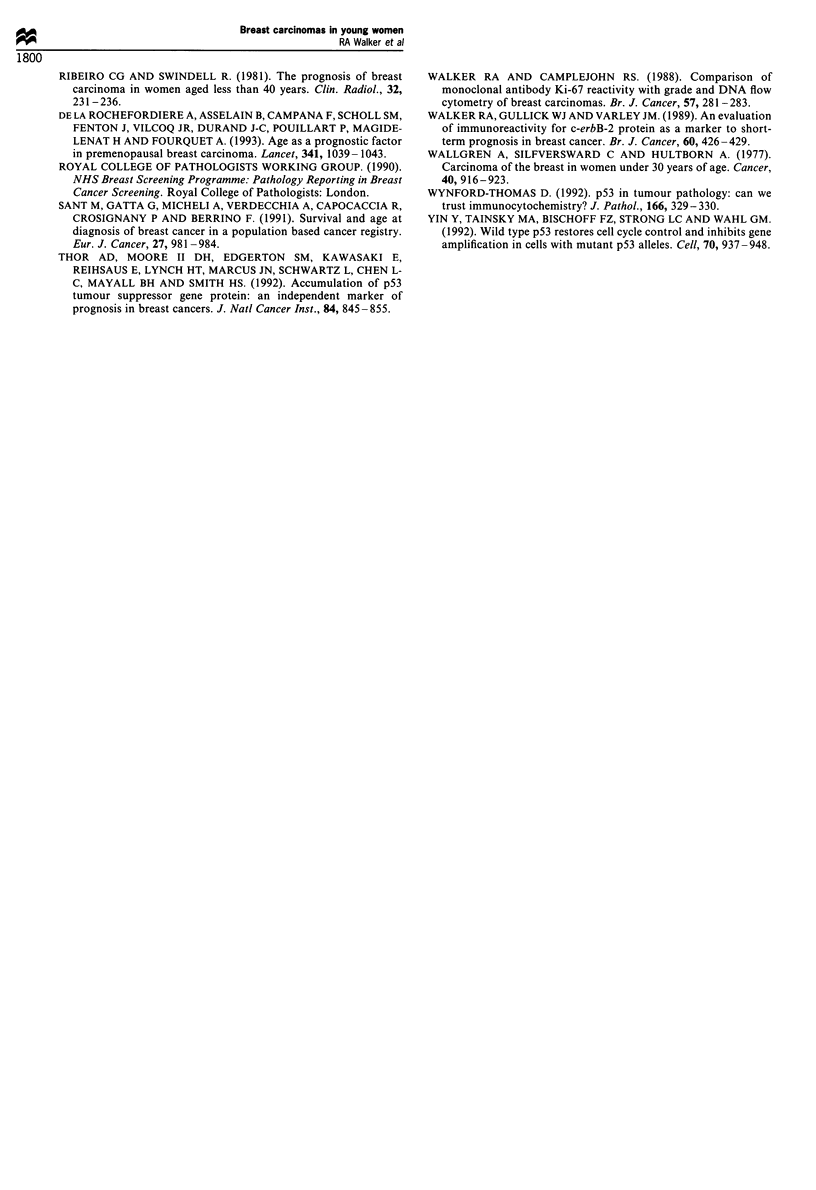# Breast carcinomas occurring in young women (< 35 years) are different.

**DOI:** 10.1038/bjc.1996.632

**Published:** 1996-12

**Authors:** R. A. Walker, E. Lees, M. B. Webb, S. J. Dearing

**Affiliations:** Breast Cancer Research Unit, University of Leicester, Glenfield Hospital, UK.

## Abstract

**Images:**


British Journal of Cancer (1996) 74, 1796-1800
© 1996 Stockton Press All rights reserved 0007-0920/96 $12.00

### Breast carcinomas occurring in young women (<35 years) are different

RA Walker, E Lees, M'B Webb and SJ Dearing

*Breast Cancer Research Unit, University of Leicester, Glenfield Hospital, Clinical Sciences, Groby Road, Leicester LE3 9QP, UK.*

**Summary** One hundred and sixty-three breast carcinomas occurring in women aged between 26 and 44 years were examined for pathological features, oestrogen and progesterone receptor status, proliferation as determined by Ki-67 labelling and the presence of c-*erb*B-2 and p53 protein, and were compared with a control group of carcinomas from women in the 50-67 years age group. Carcinomas occurring in women aged under 35 years had a significantly high incidence of being poorly differentiated and of having high proliferation rates. This group also had a significantly high incidence of p53 protein staining. Carcinomas in the under 30 years age group had a lower incidence of oestrogen and progesterone receptor positivity. No differences were found in c-*erb*B-2-positive staining between the groups. Infiltrating lobular carcinomas were only identified in women aged 40 years and over. There was a higher incidence of a family history in the 35-44 years age group (18%) than in the under 35 years age group (11%). Breast carcinomas occurring in women aged under 35 years are more aggressive. An important finding is the high incidence of p53 positivity, which may indicate genetic instability.

**Keywords:** breast cancer; young age; p53 abnormalities

There is evidence from several studies that women who develop breast cancer at a young age (either 30 years and less or 34 years and less) have lower survival rates than older patients (Wallgren *et al.*, 1977; Ribeiro and Swindell, 1981; Noyes *et al.*, 1982; Adami *et al.*, 1986; Host and Lund, 1986; Sant *et al.*, 1991; de la Rochefordiere *et al.*, 1993; Bonnier *et al.*, 1995; Chung *et al.*, 1996). The findings have not differed over a 25 year time span and have been reported from different countries. Several publications have suggested that the poorer prognosis may be related to the biological nature of the tumour (Adami *et al.*, 1986; Host and Lund, 1986; de la Rochefordiere *et al.* 1993; Chung *et al.*, 1996). Pillers (1992) combined data on histological grading from two centres and found that there was a higher frequency of poorly differentiated carcinomas in the aged 34 years and under group.

The presence or absence of various markers is associated with poorer prognosis, e.g. the overexpression of c-*erb*B-2 oncoprotein (Walker *et al.*, 1989; Gullick *et al.*, 1991), expression of p53 protein (Thor *et al.*, 1992; Barnes *et al.*, 1993) and lack of oestrogen and progesterone receptor (Foekens *et al.*, 1989; Reiner *et al.*, 1990). Higher levels of proliferation as determined by Ki-67 labelling are associated with poorer prognosis (Railo *et al.*, 1993).

In order to determine whether there is a difference in the biological nature of breast carcinomas arising in younger women, a series has been studied for a variety of markers as well as pathological features.

### Materials and methods

### *Patients*

One hundred and fifty-eight cases of invasive carcinomas were identified from the pathology files of Leicester Royal Infirmary and Glenfield Hospital, UK, and five cases were provided by Alexandra Hospital, Redditch, UK, totalling 163. Cases who had received chemotherapy and/or radiotherapy before excision of the tumour were excluded, as this may modify the immunostaining and grading. Therefore, there were no cases included which were considered clinically to be inflammatory carcinomas. All were in the age range 26-44 years, with 18 between the ages of 26 and 29 years, 30 between 30 and 34 years, 40 between 35 and 39 years and 75 between 40 and 44 years. Node status was known for 150 cases, with 84 being node-positive and 66 being node-negative. Information about family history was available for 143 cases. A group consisting of 70 symptomatic carcinomas from women aged 50-67 years for whom all marker data were available was used for comparison. Data on this group have previously been reported (Rajakariar and Walker, 1995).

### *Pathology*

Representative blocks from each case were fixed in 4% formaldehyde in saline and processed through to paraffin wax. Haematoxylin and eosin-stained sections were evaluated for type of carcinoma using the criteria published in the National Health Service (NHS) Breast Screening Guidelines (1990). Infiltrating ductal carcinomas were graded using the modified Bloom and Richardson criteria (Elston and Ellis, 1991), as recommended in the NHS Breast Screening Guidelines and in the guidelines of the Association of Directors of Anatomic and Surgical Pathology (1996).

### *Immunohistochemistry*

The following antibodies were employed:

(1) Anti-oestrogen receptor mouse monoclonal antibody (1D5) (Dako), which reacts with the N-terminal domain of the receptor. This antibody has been compared with Abbott H222 antibody applied to frozen and fixed tissue and been shown to give similar results when antigen retrieval is employed (Goulding *et al.*, 1995, unpublished observation).
(2) Anti-progesterone receptor mouse monoclonal antibody (NCL-PgR) from Novocastra.
(3) MIB-1 mouse monoclonal antibody against the Ki-67 antigen (binding site) (Cattoretti *et al.*, 1992).
(4) Polyclonal rabbit anti-p53 antiserum (CM1) (Novocastra).
(5) Mouse monoclonal anti-c-*erb*B-2 antibody (NCL CB11) from Novocastra. All secondary reagents were from Dako.

*ER, PgR and MIB-1* Formalin-fixed, paraffin-embedded sections were mounted onto slides coated with Silane (3-aminopropyltriethoxysilane, BDH) and immersed in 10 mM citric acid buffer, pH 6.0. For ER and MIB-1, the sections

Correspondence: RA Walker
Received 19 February 1995; revised 25 June 1996; accepted 1 July 1996

were exposed to three cycles, each of 5 min, of microwave irradiation using an 800 W microwave on maximum power. For PgR, two cycles were used. The antibodies were applied as follows: 1D5, 1:100 dilution in Tris-buffered saline pH 7.4; NCL PgR, 1:70 dilution; MIB-1, 1:50 dilution; all for 18 h at 4°C. Biotinylated rabbit anti-mouse immunoglobulin antiserum followed by streptavidin peroxidase was the detection system and peroxidase was localised using diaminobenzidine–hydrogen peroxide.

*c-*erb*B-2 and p53* The antibody NCL CB11 was applied at 1:80 for 18 h at 4°C, and was followed by biotinylated rabbit anti-mouse immunoglobulin antiserum and streptavidin peroxidase, as above. CM1 was applied at 1:100 for 18 h at 4°C and was followed by biotinylated swine anti-rabbit immunoglobulin antiserum and streptavidin peroxidase, with diaminobenzidine–hydrogen peroxide localisation. Controls were present in all instances with the omission of the primary antibody and the inclusion of a known positive in each staining batch.

*Evaluation* Oestrogen and progesterone receptor reactivity was categorised as negative, or as having <10%, 10–25%, 25–50%, 50–75% and >75% positive cells, with 10% being the cut-off point between positive and negative, as described previously (Rajakariar and Walker, 1995). For p53, the percentage of stained nuclei was determined with a minimum of 500 cells being counted; more than 20% of cells having moderate or strong staining was considered to be positive staining. Membrane staining of the majority of tumour cells was considered positive for c-*erb*B-2. The Ki-67 (MIB1) index was assessed by counting a minimum of 500 nuclei and calculating the percentage of stained nuclei. The results were categorised into low (<10% positive cells), medium (10–19%) and high (⩾20%) scores.

### Results

### *Pathological features*

The findings for type, grade and node status for the four categories of young breast cancers and the control group are given in Table I. There were no specialised carcinomas in the 34 years and under age groups, and no infiltrating lobular carcinomas in those patients aged 39 years and under. The distribution of types in the 40–44 years age group was similar to controls.

No well-differentiated infiltrating ductal carcinomas were found in the 34 years and less age groups, whilst the percentage of these in the other two groups and the control was similar. Sixty-nine per cent of the carcinomas from patients aged 34 years and under were poorly differentiated. There was a significant difference in the differentiation of the carcinomas of women aged 34 years and under compared with those from women aged 35–44 years ($0.02>P>0.01$, $\chi^2=8.11$, 2 d.f.) and from women aged 50–67 years ($P<0.001$, $\chi^2=14.38$, 2 d.f.) but not between women aged 35–44 years and 50–67 years ($\chi^2=4.5$, 2 d.f.).

There was a higher incidence of node-positive cases in the under 30 years age group but the numbers in this category were small.

### *Immunohistochemistry*

The overall results are shown in Table II.

There was a low incidence of oestrogen and progesterone receptor-positive carcinomas in the aged under 30 years group. There was no significant difference in the oestrogen and progesterone receptor results between the under 35 years and the 35–44 years age groups, between the under 35 years and control age groups, and between the 35–44 years and control groups.

**Table I** Histological characteristics of the young breast cancer patients and the control group

| | *Young breast cancer patients* 25–29 years | 30–34 years | 35–39 years | 40–44 years | *Cotrol* 50–67 years |
|---|---|---|---|---|---|
| Type | | | | | |
| Infiltrating ductal | 18/18 (100%) | 30/30 (100%) | 39/40 (97.5%) | 62/75 (82.5%) | 60/70 (85.5%) |
| Infiltrating lobular | 0 | 0 | 0 | 8/75 (10.5%) | 6/70 (8.5%) |
| Tubular | 0 | 0 | 0 | 3/75 (4%) | 1/70 (1.5%) |
| Mucinous | 0 | 0 | 1/40 | 2/75 (3%) | 0 |
| Medullary | 0 | 0 | 0 | 0 | 1/76 (1.5%) |
| Papillary | 0 | 0 | 0 | 0 | 2/70 (3%) |
| Grade | | | | | |
| I | 0 | 0 | 6/39 (15%) | 9/62 (14.5%) | 9/60 (15%) |
| II | 6/18 (33%) | 9/30 (30%) | 16/39 (41%) | 17/62 (27.5%) | 29/60 (48%) |
| III | 12/18 (67%) | 21/30 (70%) | 17/39 (44%) | 36/62 (58%) | 22/60 (37%) |
| Node status | | | | | |
| Positive | 11/16 (69%) | 12/26 (46%) | 25/40 (62.5%) | 36/68 (53%) | 36/64 (55%) |
| Negative | 5/16 (31%) | 14/26 (54%) | 15/40 (37.75%) | 32/68 (47%) | 28/64 (54%) |

**Table II** Incidence of receptors, c-*erb*B-2, p53 and proliferation index in relation to age

| | *Young breast cancer patients* 25–29 years | 30–34 years | 35–39 years | 40–44 years | *Cotrol* 50–67 years |
|---|---|---|---|---|---|
| Oestrogen receptor | | | | | |
| Positive | 8.18 (44%) | 17/30 (57%) | 28/40 (70%) | 37/5 (49%) | 47/70 (67%) |
| Progesterone receptor | | | | | |
| Positive | 6/18 (33%) | 11/30 (37%) | 24/40 (60%) | 31/75 (44%) | 34/70 (48.5%) |
| C-*erb*B-2 | | | | | |
| Positive | 4/18 (22%) | 6/30 (20%) | 9/40 (22.5%) | 13/75 (17%) | 12/70 (17%) |
| p53 | | | | | |
| Positive | 12/18 (67%) | 16/30 (53%) | 18/40 (45%) | 30/75 (40%) | 26/70 (37%) |
| Proliferation | | | | | |
| Low | 1/18 (6%) | 6/30 (20%) | 17/40 (42.5%) | 25/75 (33%) | 35/70 (50%) |
| Medium | 4/18 (22%) | 4/30 (13%) | 7/40 (17.5%) | 14/75 (17%) | 7/70 (10%) |
| High | 13/18 (72%) | 20/30 (67%) | 16/40 (40%) | 36/75 (50%) | 28/70 (40%) |

The range of c-*erb*B-2 positivity was 17.0–22.5%, and there were no significant differences between the different age groups.

The highest incidence of detecting p53 was in the under 30 years age group (67%) (Figure 1), with a decreasing incidence with increasing age (Figure 2). There was a significant difference between the under 35 years age group and the control group ($\chi^2 = 5.09$, 1 d.f., $0.025 > P > 0.02$), and between the under 35 years and the 35–44 years age groups ($\chi^2 = 4.27$, 1 d.f., $0.05 > P > 0.025$) but not between the 35–44 years age group and the control group.

Significant differences were found between the under 35 years age group and the control group for MIB-1 indices ($\chi^2 = 15.33$, 2 d.f., $P < 0.001$), with a higher incidence of high proliferation rates in the younger group. Differences in proliferation were also found between the under 35 years age group and the 35–44 years group ($\chi^2 = 9.17$, 2 d.f., $P = 0.01$), but not between the latter group and the control cases.

*Relationship to family history*

Information about family history was known for 143 of the women aged 44 years and younger: for 13 of the 18 women aged under 30 years, for 24 of the 30 women aged 30–34 years, for 38 of the 40 women aged 35–39 years and for 68 of the 75 women aged 40–44 years.

Two of the women under 30 years of age had a family history (15%), one who had a mother affected at age 43 years and the other whose mother was affected at age 53 years. The carcinoma from the former case was moderately differentiated and p53-positive, and that from the latter was poorly differentiated and p53-positive. Only two women between 30 and 34 years had a family history (8%), involving an aunt (premenopausal) in one case and a sister in the other. Both carcinomas were poorly differentiated and p53 positive.

The incidence of family history was higher in the 35–39 years age group (18.4%) and the 40–44 years age group (17.6%). Five women in the 35–39 years age group had a mother affected premenopausally, one woman had an aunt and a cousin affected and another had an aunt affected. Four carcinomas were moderately differentiated, three were poorly differentiated infiltrating ductal carcinomas and three were p53-positive. Six of the women in the 40–44 years age group had more than one family member affected, another two women had mothers who were affected premenopausally. Ten carcinomas were infiltrating ductal (one grade I, three grade II, six grade III), one was a tubular carcinoma and another was an infiltrating lobular carcinoma. p53 was detected in four cases, a similar incidence to this age group overall.

Figure 1 High power view of p53 staining in a poorly differentiated carcinoma from a 26-year-old woman.

**Figure 2** Percentage of cases with evidence of p53 staining in relation to age.

### Discussion

The study has shown that although there clearly are differences in the carcinomas arising in women aged under 35 years, the carcinomas arising in women aged 35–44 years are not significantly different from those occurring in women aged 50–67 years. This emphasises the importance of the subdivision of the under 50 years age group in any studies that consider prognostic factors.

The differences in the carcinomas encompass both pathological and biological features, although the two are probably related. The high incidence of poorly differentiated carcinomas in the under 35 years age group has been reported by others (Pillers, 1992). It is striking that no well-differentiated carcinomas were found in this age group and that the differentiation of carcinomas in the 35 and over age group was not significantly different from the older age group. The 35–44 years age group had a higher incidence of family history than the under 35 years age group and showed no particular relationship to tumour type, grade and p53 status.

Both oestrogen receptor status and proliferation correlate with differentiation. The presence of oestrogen receptor is associated with better differentiation (Bruun Rasmussen *et al.*, 1981). It is therefore not surprising that there is a low incidence of oestrogen receptor positivity in the 29 years and younger age group. This has also been reported by Albain *et al.* (1994). However, 57% of the carcinomas in the 30–34 years age group were oestrogen receptor positive, a value not significantly different from the other age groups. This suggests that there are other factors determining the oestrogen receptor status as 70% of carcinomas in this group were poorly differentiated. Proliferation as determined by Ki-67 antigen detection relates to tumour differentiation (Walker and Camplejohn, 1988), and high levels of Ki-67 labelling were seen in the two age groups with a high incidence of poorer differentiation. A high S-phase fraction was found in 60% or more of carcinomas from women 35 years and younger (Albain *et al.*, 1994), which is similar to the findings in this study for Ki-67.

Infiltrating lobular carcinomas were only identified in women aged 40 years or more. Marcus *et al.* (1994) have reviewed the literature with regard to the pathology of early onset of breast carcinoma. They considered that there was a significant trend for less invasive lobular carcinoma in the younger age group and noted the effect to be most prominent in the 20–29 year age group. However, such a clear cut-off point at 40 years has not been reported by others.

Although c-*erb*B-2 expression has been related to poorer differentiation (Walker *et al*., 1989; Allred *et al*., 1992), there was no difference in expression between the different age groups and the control. Allred *et al*. (1992) found a higher incidence of c-*erb*B-2 expression in cases of infiltrating ductal carcinoma with associated ductal carcinoma *in situ*, and which they found more frequently in a younger age group. However, their group (Albain *et al*., 1994) found no significant difference in expression across age groups.

Apart from differentiation and proliferation, the one marker which was significantly different between the under 35 years age group and the other age groups was p53, with a high incidence of 67% positive cases in the under 30 years age group. The presence of p53 protein does not necessarily imply that there is a mutation as other factors can lead to stabilisation and hence reactivity (Wynford-Thomas, 1992). Both p53 protein staining and mutation have been associated with poorer differentiation and oestrogen and progesterone receptor-negative tumours (Walker *et al*., 1991; Mazars *et al*., 1992; Thor *et al*., 1992; Barnes *et al*., 1993; Jacquemier *et al*., 1994). When age has been considered, it has usually involved the subdivision of women into under or over 50 years of age, and no significant difference has been found. Caleffi *et al*. (1994) did find a significantly higher incidence of p53 mutations in younger women, using 45 years of age as the cut-off point. Albain *et al*. (1994) also reported a striking incidence of p53 protein in the 35 years and under age group.

Several studies have implicated p53 protein in the $G_1$–S arrest which occurs in response to DNA damage (Kuerbitz *et al*., 1992; Yin *et al*., 1992). p53 activates a $M_r$ 21 000 protein (Cip/WAF1/SD1) which inhibits the activity of cyclin-dependent kinases and thus induces arrest in $G_1$ or apoptosis (El-Deiry *et al*., 1994). Cells with abnormal p53 do not activate p21 and do not show normal $G_1$ arrest which is necessary for repair after exposure to DNA-damaging agents. In a study of 183 breast carcinomas, Eyfjord *et al*. (1995) found a significant association between p53 abnormalities and genetic instability.

It will be of particular interest and importance to analyse the breast carcinomas occurring in the young age group to determine whether there are any common p53 abnormalities and whether there are any associated DNA repair defects.

**Acknowledgements**
We are grateful to Mrs Margaret Hornby for typing the manuscript and to Dr Louise Brown, Department of Histopathology, Alexandra Hospital, Redditch, UK, for providing some of the cases.

### References

ADAMI HO, MALKER B, HOLMBERG L, PERSONN I AND STONE B. (1986). The relation between survival and age at diagnosis in breast cancer. *New Eng. J. Med.*, **315**, 559–563.

ALBAIN KS, ALLRED DC AND CLARK GM. (1994). Breast cancer outcome and predictors of outcome: are there age differentials? *Monogr. Natl Cancer Trust*, **16**, 35–42.

ALLRED DC, CLARK GM, MOLINA R, TANDON AK, SCHNITT SJ, GILCHRIST KW, OSBORNE CK, TORMEY DC AND MCGUIRE WL. (1992). Over expression of HER-2/*neu* and its relationship with other prognostic factors change during the progression of in-situ to invasive breast cancer. *Hum. Pathol.*, **23**, 974–979.

ASSOCIATION OF DIRECTORS OF ANATOMIC AND SURGICAL PATHOLOGY. (1996). Recommendations for the reporting of breast carcinoma. *Human Pathol.*, **27**, 220–224.

BARNES DM, DUBLIN EA, FISHER CJ, LEVISON DA AND MILLIS RR. (1993). Immunohistochemical detection of p53 protein in mammary carcinoma: an important new independent indication of prognosis? *Hum. Pathol.*, **24**, 469–476.

BONNIER P, ROMAIN S, CHARPIN C, LEJEUNE C, TUBIANA N, MARTIN P-M AND PIANA L. (1995). Age as a prognostic factor in breast cancer: relationship to pathologic and biologic features. *Int. J. Cancer*, **62**, 138–144.

BRUNN RASMUSSEN B, ROSE C, THORPE SM, HOU-JENSEN K, DAEHNFELDT JL AND PALSHOF T. (1981). Histopathological characteristics and oestrogen receptor content in primary breast carcinoma. *Virchows Arch. Pathol. Anat.*, **390**, 347–351.

CALEFFI M, TEAGUE MW, JENSEN RA, VNENCAK-JONES CL, DUPONT WD AND PARL FF. (1994). p53 gene mutations and steroid receptor status in breast cancer. *Cancer*, **73**, 2147–2156.

CATTORETTI G, BECKER MHG, KEY G, DUCHROW M, SCHLUTER C, GALLE J AND GERDES J. (1992). Monoclonal antibodies against recombinant parts of the Ki-67 antigen (M1B-1 and M1B-3) detect proliferating cells in microwave processed formalin-fixed paraffin sections. *J. Pathol.*, **168**, 357–363.

CHUNG M, CHANG HR, BLAND KI AND WANEBO HJ. (1996). Younger women with breast carcinoma have a poorer prognosis than older women. *Cancer*, **77**, 97–103.

EL-DEIRY WS, HARPER JW, O'CONNER PM, VELCULESCO VE, CANMAN CE, JACKMAN J, PIETENPOL JA, BURREL M, HILL DE, WANG Y, WIMAN KG, MERCER WE, KASTAN MB, KOHN KW, ELLEDGE SJ, KINZIVER KW AND VOGELSTEIN B. (1994). WAF1/CIP1 is induced in p53-mediated G1 arrest and apoptosis. *Cancer Res.*, **54**, 1169–1174.

ELSTON CW AND ELLIS IO. (1991). Pathological prognostic factors in breast cancer. I. The value of histological grade in breast cancer: experience from a large study with long term follow-up. *Histopathology*, **19**, 403–410.

EYFJORD JE, THORLACIUS S, STEINARSDOTTIR M, VALGARDSDOTTIR R, OGMUNDSDOTTIR HM AND ANAMTHAWAT-JONSSON K. (1995). p53 abnormalities and genomic instability in primary human breast cancers. *Cancer Res.*, **55**, 646–651.

FOEKENS JA, PORTENGEN H, VAN PUTTEN WLJ, PETERS HA, KRITJNEN HLJM, ALEXIEVA-FIGUSCH J AND KLIJN JGM. (1989). Prognostic value of estrogen and progesterone receptors measured by enzyme immunoassays in human breast tumour cytosols. *Cancer Res.*, **49**, 5823–5828.

GULLICK WJ, LOVE SB, WRIGHT C, BARNES DM, GUSTERSON B, HARRIS AL AND ALTMAN DG. (1991). C-*erb*B-2 protein over expression in breast cancer is a risk factor in patients with involved and uninvolved lymph nodes. *Br. J. Cancer*, **63**, 434–438.

HOST H AND LUND E. (1986). Age as a prognostic factor in breast cancer. *Cancer*, **57**, 2217–2221.

JACQUEMIER J, MOLES JP, PENAULT-LLORCA F, ADELAIDE J, TORRENTE M, VIENS P, BIRNBAUM B AND THEILLET C. (1994). p53 immunohistochemical analysis in breast cancer with four monoclonal antibodies: comparison of staining and PCR-SSCP results. *Br. J. Cancer*, **69**, 846–852.

KUERBITZ SJ, PLUNKETT BS, WALSH WV AND KASTAN MB. (1992). Wild-type 53 is a cell cycle checkpoint determinant following irradiation. *Proc. Natl Acad. Sci. USA*, **89**, 7491–7495.

MARCUS JN, WATSON P, PAGE DL AND LYNCH HT. (1994). Pathology and heredity of breast cancer in younger women. *Monogr. Natl Cancer Inst.*, **16**, 23–34.

MAZARS R, SPINARD IL, BENCHEIKH M, SIMONY-LAFONTAINE J, JEANTEUR P AND THEILLET C. (1992). p53 mutations occur in aggressive breast cancer. *Cancer Res.*, **52**, 3918–3923.

NOYES RD, SPANOS WJ AND MONTAGUE ED. (1982). Breast cancer in women aged 30 and under. *Cancer*, **49**, 1302–1307.

PILLERS EMK. (1992). Histological grade of breast cancer in younger women. *Lancet*, **339**, 1483.

RAILO M, NORDLING S, VON BOGUSLAWKSKY K, LEIVONEN M, KYLLONEN L AND VONSMITTEN K. (1993). Prognostic value of Ki-67 immunolabelling in primary operable breast cancer. *Br. J. Cancer*, **68**, 579–583.

RAJAKARIAR R AND WALKER RA. (1995). Pathological and biological features of mammographically detected invasive breast carcinomas. *Br. J. Cancer*, **71**, 150–154.

REINER A, NEUMEISTER B, SPONA J, REINER G, SCHEMPER M AND JAKESZ R. (1990). Immunocytochemical localisation of estrogen progesterone receptor and prognosis in human primary breast cancer. *Cancer Res.*, **32**, 7057–7061.

RIBEIRO CG AND SWINDELL R. (1981). The prognosis of breast carcinoma in women aged less than 40 years. *Clin. Radiol.*, **32**, 231-236.

DE LA ROCHEFORDIERE A, ASSELAIN B, CAMPANA F, SCHOLL SM, FENTON J, VILCOQ JR, DURAND J-C, POUILLART P, MAGIDE-LENAT H AND FOURQUET A. (1993). Age as a prognostic factor in premenopausal breast carcinoma. *Lancet*, **341**, 1039-1043.

ROYAL COLLEGE OF PATHOLOGISTS WORKING GROUP. (1990). *NHS Breast Screening Programme: Pathology Reporting in Breast Cancer Screening*. Royal College of Pathologists: London.

SANT M, GATTA G, MICHELI A, VERDECCHIA A, CAPOCACCIA R, CROSIGNANY P AND BERRINO F. (1991). Survival and age at diagnosis of breast cancer in a population based cancer registry. *Eur. J. Cancer*, **27**, 981-984.

THOR AD, MOORE II DH, EDGERTON SM, KAWASAKI E, REIHSAUS E, LYNCH HT, MARCUS JN, SCHWARTZ L, CHEN L-C, MAYALL BH AND SMITH HS. (1992). Accumulation of p53 tumour suppressor gene protein: an independent marker of prognosis in breast cancers. *J. Natl Cancer Inst.*, **84**, 845-855.

WALKER RA AND CAMPLEJOHN RS. (1988). Comparison of monoclonal antibody Ki-67 reactivity with grade and DNA flow cytometry of breast carcinomas. *Br. J. Cancer*, **57**, 281-283.

WALKER RA, GULLICK WJ AND VARLEY JM. (1989). An evaluation of immunoreactivity for c-*erb*B-2 protein as a marker to short-term prognosis in breast cancer. *Br. J. Cancer*, **60**, 426-429.

WALLGREN A, SILFVERSWARD C AND HULTBORN A. (1977). Carcinoma of the breast in women under 30 years of age. *Cancer*, **40**, 916-923.

WYNFORD-THOMAS D. (1992). p53 in tumour pathology: can we trust immunocytochemistry? *J. Pathol.*, **166**, 329-330.

YIN Y, TAINSKY MA, BISCHOFF FZ, STRONG LC AND WAHL GM. (1992). Wild type p53 restores cell cycle control and inhibits gene amplification in cells with mutant p53 alleles. *Cell*, **70**, 937-948.